# A Cross-Disciplinary View of Testing and Bioinformatic Analysis of SARS-CoV-2 and Other Human Respiratory Viruses in Pandemic Settings

**DOI:** 10.1109/ACCESS.2021.3133417

**Published:** 2021-12-06

**Authors:** Md Arafat Hossain, Barbara Brito-Rodriguez, Lisa M. Sedger, John Canning

**Affiliations:** Department of Electrical and Electronic EngineeringKhulna University of Engineering & Technology Khulna 9203 Bangladesh; Faculty of Sciencei3 Institute, University of Technology Sydney (UTS) Sydney NSW 2007 Australia; Faculty of ScienceUniversity of Technology Sydney (UTS) Sydney NSW 2007 Australia; interdisciplinary Photonic Laboratories (iPL), Global Big Data Technologies Centre (GBDTC), Faculty of Engineering and Information TechnologyUniversity of Technology Sydney (UTS) Sydney NSW 2007 Australia

**Keywords:** Antibody test, antigen test, bioinformatics, COVID-19, Internet-of-Things, lab-in-a-phone, LAMP test, point-of-care test, PCR, SARS-CoV

## Abstract

The SARS-Coronavirus-2 (SARS-CoV-2) infectious disease, COVID-19, has spread rapidly, resulting in a global pandemic with significant mortality. The combination of early diagnosis via rapid screening, contact tracing, social distancing and quarantine has helped to control the pandemic. The absence of real time response and diagnosis is a crucial technology shortfall and is a key reason why current contact tracing methods are inadequate to control spread. In contrast, current information technology combined with a new generation of near-real time tests offers consumer-engaged smartphone-based “lab-in-a-phone” internet-of-things (IoT) connected devices that provide increased pandemic monitoring. This review brings together key aspects required to create an entire global diagnostic ecosystem. Cross-disciplinary understanding and integration of both mechanisms and technologies for effective detection, incidence mapping and disease containment in near real-time is summarized. Available measures to monitor and/or sterilize surfaces, next-generation laboratory and smartphone-based diagnostic approaches can be brought together and networked for instant global monitoring that informs Public Health policy. Cloud-based analysis enabling real-time mapping will enable future pandemic control, drive the suppression and elimination of disease spread, saving millions of lives globally. A new paradigm is introduced – scaled and multiple diagnostics for mapping and spreading of a pandemic rather than traditional accumulation of individual measurements. This can do away with the need for ultra-precise and ultra-accurate analysis by taking mass measurements that can relax tolerances and build resilience through networked analytics and informatics, the basis for novel swarm diagnostics. These include addressing ethical standards, local, national and international collaborative engagement, multidisciplinary and analytical measurements and standards, and data handling and storage protocols.

## I. Introduction

A novel respiratory disease caused by the severe acute respiratory syndrome Coronavirus-2 (SARS-CoV-2) was first reported in Wuhan China, in December 2019 [1]. It was defined by the World Health Organization (WHO) as “coronavirus infectious disease of 2019” or COVID-19 [2]. SARS-CoV-2 virus spread quickly within China and internationally creating a global pandemic: by November 2021, WHO estimates that at least 263 million people worldwide have been infected [2]. This markedly contrasts with other human coronaviruses that circulate frequently in humans causing upper respiratory tract infection but with milder symptoms [3]. SARS-CoV-2 infects upper and lower respiratory tract tissues and further spreads to other tissues generating diverse pathologies. Systemic inflammatory immune responses is frequently observed where high levels of inflammatory cytokines, known as a cytokine storm, are generated [4]. Thus, the pathobiology of SARS-CoV-2 infection is complex. Blood clotting events also occur, and there are reports of an autoimmune response in infected children that resembles Kawasaki disease [5]. Since the virus has been spreading through a naïve immune system, the infection has evolved and changed over time. Initially COVID-19 was particularly virulent in people over 50 years of age, especially in males [6]. However, more recent variants are increasingly infectious and virulent in younger people [7]. For example, the delta and omicron variants are estimated to be several times more infectious than the virus found originally in Wuhan. Fortunately, optimal treatments in different patient cohorts are resulting in higher survival rates.

An understanding of the major transmission mechanisms of SARS-CoV-2 has helped to mandate partially effective infection control measures. The virus is primarily spread by respiratory droplet and aerosol particles [8], leading to recommendations for health care workers to use N95 masks, and for general masking, social distancing and home quarantine for the wider population. The sustainability of the SARS-CoV-2 virus on surfaces, impacts on the choice of control measures being implemented. Nevertheless, studies investigating the likely origins of the SARS-CoV-2 are based primarily on nucleic acid homology. These show high similarity with existing bat coronaviruses suggestive of a spill-over event from bats to humans and/or through an intermediate animal species [9]–[11]. The nature of this spillover event is highly contested.

Traditional laboratory-based diagnostics such as reverse transcriptase-polymerase chain reaction (RT-PCR) generally offers accurate results. However, they require specific equipment, laboratory expertise and time to perform to confirm positive results. Unfortunately, rapid accurate diagnostic tests for COVID-19 underpin the success of viral pandemic infection control measures. In Europe, the US and Singapore, rapid diagnostic COVID-19 tests are therefore widely deployed to help detect the virus and control transmission through infected individuals. For various reasons, other countries have been more cautious in the use of rapid-testing devices. Despite reticence over accuracy and other factors, real-time (rapid) diagnostics for SARS-CoV-2 virus detection are the first stage of a new future paradigm of interconnected diagnostic mapping to detect and contain pandemics in near to real-time. Coupled with ubiquitous consumer technologies, such as smartphones-based technologies, the development and integration of novel biosensors, permits high speed wireless connectivity for globally interconnected infectious disease incidence monitoring.

Rapid detection tests for SARS-CoV-2 are increasingly available globally (for reviews see [12]–[16]). Whilst existing literature focuses largely on traditional laboratory-based nucleic acid- or antibody-based COVID-19 tests, with various modifications, there has been alternative advances that can offer faster response and wider connectivity. These include surface and wearable sensor technologies, and lab-in-a-phone type platforms, that together with network diagnostics such as cloud-based informatics, creates a much-improved global monitoring capability. Here, we provide a cross-disciplinary review of current technologies, bridging the gap between biomedical technologies and IoT engineering. This review will: (1) discuss the major mechanisms of SARS-CoV-2 transmission, highlighting the important role of water and its impact on viral spread and detection, (2) review the range of detection and diagnostics platforms available as point-of-care (POC) tests (for clinicians), (3) describe the main smartphone-based “lab-on-a-phone” technologies that can read and analyze POC tests, and (4) discuss the integration of networked field diagnostics where millions of smartphone instruments can be interconnected for enhanced instant and resilient health informatics for detection, mapping and containment of pandemic pathogens. The concept of novel super instruments based on swarm diagnostics is described. Finally, we draw attention to issues raised by such interconnected technologies including ethics around patient information, privacy rights and confidentiality of test result data and its use locally and globally.

## II. Testing in the Context of Respiratory Virus Transmission

The estimated total number of COVID-19 cases is always dependent on, one, testing rates and, two, the percentage of both symptomatic and asymptomatic infected people being tested. For example, asymptomatic people are less likely to be submitting themselves for testing despite being infectious because they are unlikely to consider themselves infected when any symptom is mild. The incubation period for SARS-CoV-2 to produce symptoms of COVID-19 are usually 4–5 days from the date of infection, but asymptomatic infection can start earlier and extend as much as 14 days or more. Nevertheless, up to this point 97.5% of those who experience symptoms tend to do so within 11.5 days of infection [17], [18] and many well before this.

Any test can yield a false negative test result (albeit often at very low rates) and some infected individuals remain asymptomatic and/or harbour very low levels of virus that are below the detection limit for several days post-infection [19]. Each of these situations permits infectious people to continue to spread the virus. In the context of a pandemic, the factors that influence viral spread include both the nature of the infective agent (in this case an RNA virus) and the environment it occupies. For example, indoor/outdoor air in various moisture levels, on different surface types, before or after surface cleaning, all factors that directly influence infectious transmission.

### A. Water Vapour, Droplets and Aerosols – the Role of Water

Emitted airborne water such as atmospheric droplets are relatively large and generally fall to the ground or onto surfaces within 2 m where they can survive on surfaces for several days or longer [20]. “Physical distancing” aims to avoid exposure to these infectious droplets. Regular hand washing and wearing masks is also an effective (though not 100 %) measure that reduces droplet-based infection arising from surface fomites and aerosol. The practical effectiveness of distancing and masking tends to wane once the pandemic is perceived as non-serious or being “under control” and complacency sets in. Importantly, smaller droplets or aerosols are $\leq \!1 ~\mu \text{m}$ in size and present in human breath – for the recent delta strain this has become the main vector of transmission requiring that both effective masking and distancing be rigidly maintained.

Aerosols are spherical due to the surface tension that arises from the dipole alignment of water, both actual and instantaneous (or van der Waals), on the droplet surface. This creates a quasi-self-aligned charge-resistant surface that resists evaporation and extends the drop lifetime. Consequently, aerosols can be transmitted well beyond 2 m, presenting an almost intractable challenge – for example, passing infected joggers can potentially infect dozens of people with an aerosol reach of tens of meters even outdoors. SARS-CoV-2 is capable of aerosol spread, particularly the delta variant [21]. One approach to combat this in buildings is to ensure sufficient ventilation, dispersing and accelerating drying of the aerosol droplet. Once the water content is removed, the membrane-enveloped virus is generally denatured or rendered inactive. The spike proteins used for attachment to cells have protruding proteins that may be lost (together with the lipid membrane) or rendered non-functional by virtue of the structural impact of dipole dehydration. UV irradiation can also disrupt the virus in the droplets [14], a factor that may explain why viral spread in Australia has been noticeably low despite huge congregations of complacent individuals at beaches.

A respiratory virus particle can be present both within water droplets and in or on aerosols – depending on its charge. It is not known whether the virus can survive for longer *within* the droplet, or *on* the surface of the droplet but an air-water interface is charged differently to the droplet interior surface interface [22]. Other component substances such as mucus means that the drop is never actually an “idealized” water drop, but a complex drop, with variable properties that affect droplet lifetimes and drop size. Additional lifetime factors include rapid drying of a surface that accelerates droplet denaturing, or humidity that will prolong droplet longevity and virus viability. In this regard, factors are being considered that alter the interaction of the virus particle with the epithelium. Certain molecules such as carrageenan can be produced in nasal sprays to inhibit droplets from attaching to the nasal passageway by virtue of mimicking cell surface heparan sulphate proteoglycans. Clinical trials are currently assessing if carrageenan-based nasal and throat sprays reduce SARS-CoV-2 infection in at-risk health care workers [23]. Similarly, hypromellose (hydroxypropylmethylcellulose) has been used for the controlled delivery of hydrophilic drug materials [24] and is also being considered for its potential to inhibit SARS-CoV-2 [25].

Whether in droplets, or aerosols, masks function to protect from respiratory spread viruses. Masks should ideally be comprised of three layers. The innermost layer limits expelled virus (from the infected person) where electret generation draws water away from the surfaces and helps to capture smaller drops that might otherwise pass through the mask pores, trapping some of this at the inner layer. The outer layer acts as a hydrophobic surface repellent (after charging its electrostatic surface – similarly exploiting the attraction/repulsion of the aerosol’s charge). Thus, masks reduce the potential reception of virus-laden droplets by the receiver. There is also potential to add antiviral disinfectants within the central layers to disrupt the viral material and render it uninfectious [26].

### B. Surface Spread of Fomites

Contaminated surfaces occur where water droplets or aerosols reach surfaces. Depending on whether a surface is hydrophilic or hydrophobic, a virus that is present within water vapour is deposited onto the surface. Viruses only replicate within an infected host cell (in this case a human or animal cells) but can exist, without replicating, on a surface for days or longer [20]. As surfaces differ, so too does the potential for long- or short-term virus livability. For example, smooth solid surfaces such as stainless steel, glass or clear plastic face shields have different hydrophobic and hydrophilic properties to rough surfaces. At its simplest, this impacts the droplet drying rate and therefore the survivability of the virus particle.

Water assembly processes at the surface-water interface and van der Waals electrostatic binding are factors that determine the longevity of a water droplet or aerosol on that surface. In fact, water molecules can form stable atomic level interfacial surface layers and exist there for long periods, even days, at room temperature, well after the bulk of the droplet has evaporated [27]. However, these water layers can be rapidly removed by heating to ~ 40 °C [22], faster at 70 °C [28]. Therefore, high temperature industrial blow dryers could be used to both quickly dry and denature viruses that are present on surfaces, preventing spread of the aerosol. An alternative approach is to treat the surface to attract water droplets, further spreading them over the surface, or within a porous surface coating, to aid drying. Drying quickly denatures the virus on the surface. The addition of an antiviral or antibiotic disinfectant to a surface coating can render the virus quickly uninfectious to human cells. Mini protein domain reagents could even theoretically be used as a viral attractant trap, especially on critical surfaces such as stainless-steel hospital door handles. A consideration is ensuring potency of the disinfectant layer after repeated surface cleaning. Nevertheless, several different protein micro-domain attractors could be combined together in a single application mixture for absorption and denaturation of different human respiratory-spread viruses.

Although this review focuses on respiratory viruses, both SARS-CoV-1 and −2 infected humans also shed virus in their faeces [6]. Hence faecal derived droplet/aerosols provide an avenue for fomite-based human-human transmission of the virus [29]. This was evident in the earlier SARS-CoV-1 outbreak, where aerosolized virus-contaminated faecal content permitted the spread of the virus within the Amoy Gardens high-rise apartments in Hong Kong. Plumbing designs and engineering for the use of a water trap (disconnecting the continuity of the sewage drainage and floor drain systems) prevents faecal-aerosol spread of viruses [30]. The faecal presence of virus includes both infectious viral and viral remnants. In Australia, and other places around the world, sewage surveillance is used to identify community persistence of the virus, even amidst a period when zero daily new cases are recorded [31]. Indeed, sewage detection of the virus can be the first indicator of the spread of infections of coronaviruses within a new community population.

## III. Diagnostics

In considering detection of pandemic spread of a respiratory pathogen, particularly viruses, one must understand the methods of viral testing and surveillance. The next section reviews the principles of laboratory and point-of care (POC) detection testing for human respiratory viruses, focusing particularly on SAR-CoV-2 and new detection technologies.

### A. Virus Nucleic Acid Tests

**FIGURE 1.**
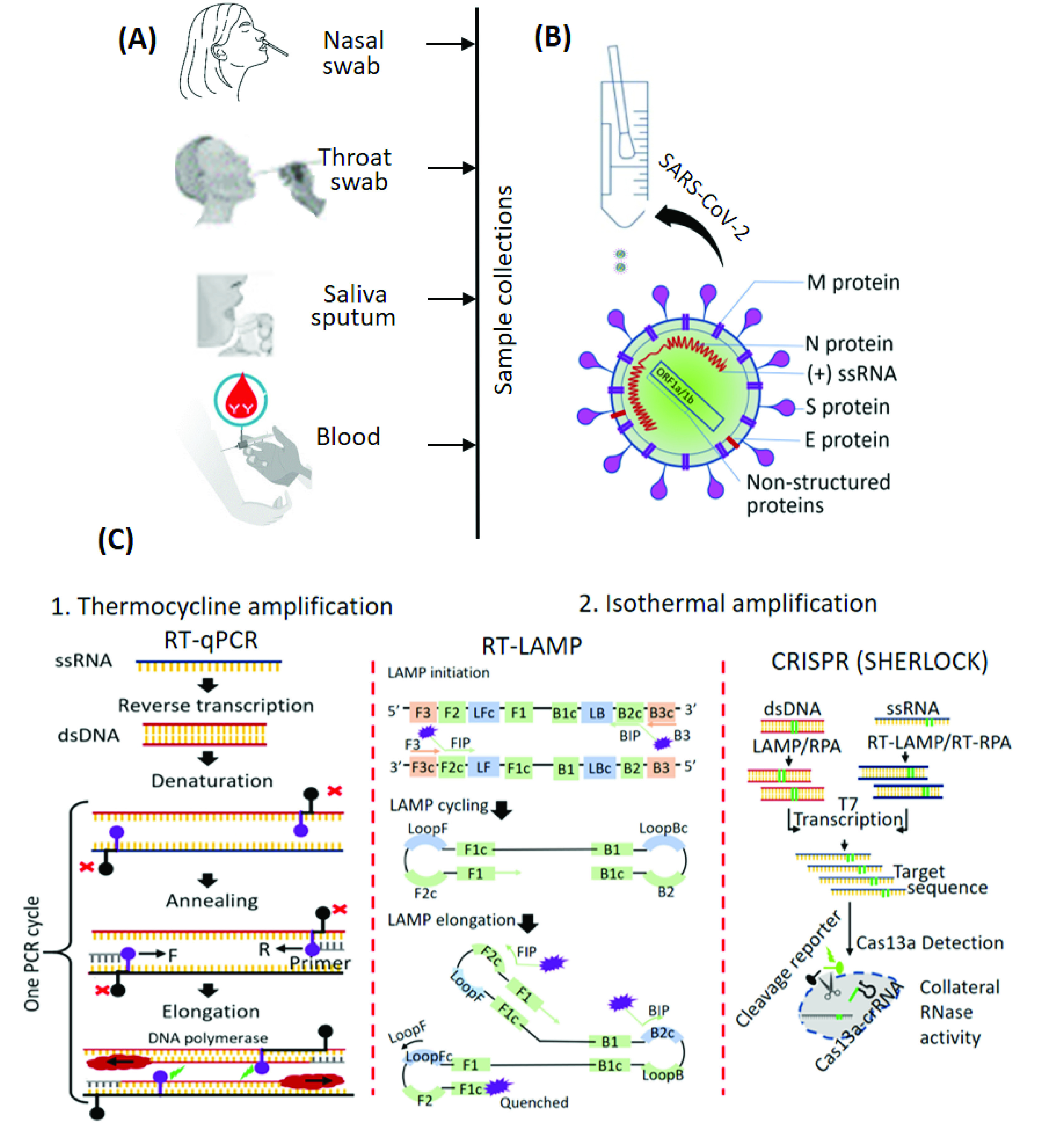
Schematic of current diagnostic methods for viral nucleic acid detection. (A) Sampling from suspected cases or patients produces the test specimen. (B) Test samples and the structure of the SARS-CoV-2 virus. (C) Mechanisms of the COVID-19 diagnosis using nucleic acid-based tests: RT-PCR, RT-LAMP & the CRISPR (SHERLOCK) systems.

Nucleic acid tests directly detect the presence of a virus genome: either DNA or RNA. For SARS-CoV-2, or influenza viruses the genomes are RNA, which can easily be extracted from respiratory secretions collected on nasopharyngeal and throat swabs (Fig 1A and B) [32]. The samples are analyzed using three main methods:

#### 1) Polymerase Chain Reaction (PCR)

A standard PCR assay copies and amplifies the nucleic acid and is the “gold standard” for virus detection. For RNA viruses, the process starts with a reverse transcription reaction; a test step that converts a single strand of extracted viral genome RNA into a complimentary sequence of single stranded DNA (ssDNA). The DNA template is amplified generating a double stranded DNA (dsDNA) PCR product (Fig 1C). In PCR, repeated iterations, amplify the cDNA up to 2^40^ times (using 40 or more cycles) producing high yields of the cDNA PCR product, that are visualized by fluorescent intercalating dyes or fluorescently-conjugated DNA sequence-specific oligonucleotide probes [33], [34]. For each PCR “cycle”, a temperature of $T = 95\,\,^{\circ }\text{C}$ denaturing step is followed by oligonucleotide primer annealing step of $T = 50$-60 °C. Here, the viral sequence specific DNA oligomer anneals to the targeted genome sequence and the viral nucleic acid is amplified by a DNA polymerase. The PCR product – a cDNA copy of the segment of viral genome template - can then be sequenced (if required). By repeating the PCR cycling process up to 40 times, logarithmic amplification leads to high sensitivity. The primer sequences confer high test specificity whilst the fluorescence can provide quantitative estimates of viral load.

PCR tests require skilled laboratory scientists and a thermal cycler (equipment). In times of pandemic spread of the virus, the PCR detection test rates must be high in order to provide useful community-level information about infection incidence. To address this in practice, patient samples are routinely pooled (usually 5–10 patient samples/pool) and the PCR is run on the pooled sample. Confirmation of a positive result is then achieved by first re-extracting the nucleic acid from the individual patient samples, and usually between 3–5 separate PCRs are then performed – repeating the original sequence-specific PCR, as well performing several other sequence-specific PCRs (targeting other viral genome segments). Thus, a true positive result is determined based on the positive detection of a minimum of 3 positive PCRs of viral nucleic acid [34]. A negative PCR reaction without any extracted viral nucleic acid is always included in the test run because this confirms that a positive reaction is not due to reagent contamination. However, the obvious limitation to PCR testing is the need for the virus-nucleic acid specific oligonucleotide primers, and hence the virus must first be characterized to some extent, such that the virus genome, or a partial sequence of it, is determined $a$ *priori*. Thus, metagenomics nucleic acid sequence is first obtained with random primers and the overlapping sequences assembled.

A range of commercially available RT-PCR test kits are currently available all human respiratory viruses: adenovirus, coronavirus SARS-CoV-2 and influenza, respiratory syncytial virus and rhinovirus. In practice, however, when a new virus emerges the PCR test is developed “in house” within a clinical microbiology laboratory and the primer sequences quickly shared internationally, rapidly establishing a standardized nucleic acid detection assay.

#### 2) Loop-Mediated Isothermal Amplification (Lamp)

An adaptation of the standard PCR assay is the LAMP method that amplifies the viral nucleic acid at a constant temperature (removing the need for a thermocycler) (Fig 1C). LAMP uses DNA polymerase with high strand displacement and replication activity, and two “inner” primers and “outer” primers that each recognizes separate regions on the target nucleic acid. The LAMP reaction is complete when all primers are correctly annealed to the template, achieving a high specificity reaction, reportedly with a low rate of false positive results [35]. Of note, the amount of DNA produced in LAMP reactions is higher than many conventional PCR reactions and the results can be visible to the naked eye [36]–[38]. The detection sensitivity is improved by a customized chip to integrate rapid nucleic acid extraction based on an immiscible phase filtration method assisted by surface tension and digital isothermal amplification [39].

#### 3) CRISPR-Based Sherlock System

Another molecular viral nucleic acid detection method utilizes the clustered, regularly interspaced, short palindromic repeats (CRISPR) family of enzymes, such as Cas9, Cas12 and Cas13. Such methods are already adapted for the detection of SARS-CoV-2 nucleic acid [40], [41]. This method relies on the endonuclease activity of the CRISPR enzyme after it binds to a specific target RNA via a guiding (programmable) RNA. The release of a reporter molecule yields a detectable signal – providing utility as a diagnostic. Specific high-sensitivity enzymatic reporter unlocking or “SHERLOCK” is one such assay (Fig. 1C), reportedly achieving attomolar sensitivity and single-based mismatched nucleic acid specificity [42] – of significant benefit when strain (mutation) based discrimination is required. Moreover, this assay can be read using a dipstick with the result generated within 40 minutes [43]. Recently, SARS-CoV-2 was detected from CRISPR-Cas13a assay of nasal swab RNA and read using a smartphone microscope [44].

### B. Nucleic ACID Analysis, Bioinformatics & Global Surveillance of Pandemics

**FIGURE 2.**
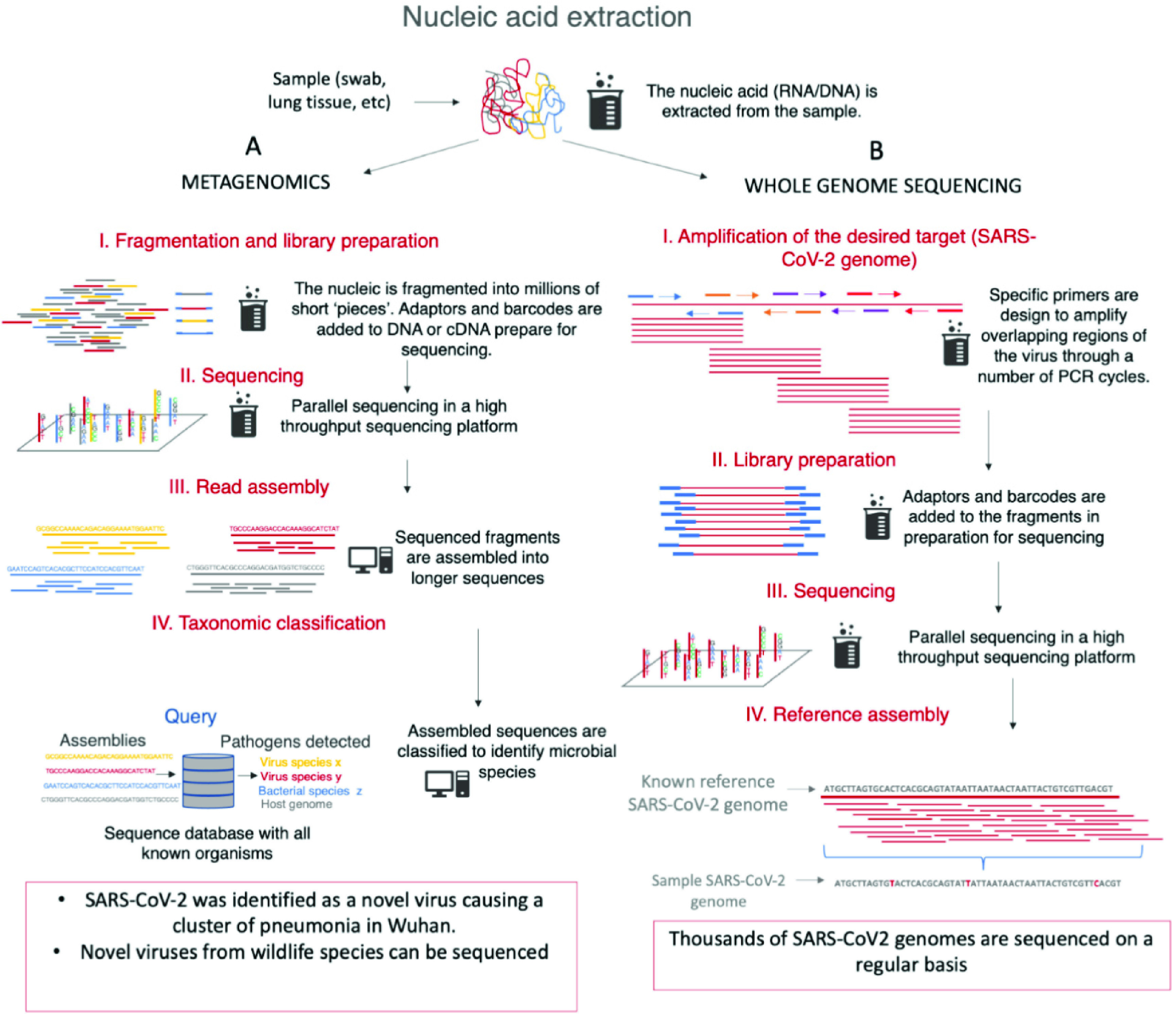
Next generation sequencing technologies applications during the SARS-CoV-2 pandemic. A. Metagenomics approaches used at the beginning of the SARS-CoV-2 epidemic to investigate the causal agent of a cluster of “novel” pneumonia cases, in Wuhan, China. Extraction of nucleic acid, fragmenting and amplifying the nucleic acid fragments, then adding “adaptors” that permit the fragments bind to a flow cell in the NGS platform. All fragments are sequenced in parallel, and detected by different fluorescent signals emitted by the four nucleotides: A, T, G, C. The sequence for all fragments is assembled to generate a contiguous “reference” sequence. The sequences are then queried against existing reference databases. This permits the taxonomic classification and identification of genomic species. B. Whole genome sequence of SARS-CoV-2 also begins with nucleic acid extractions, then amplifying selected targeted genome regions with specific oligonucleotide primers that bind to overlapping regions of the viral genome. This PCR product is sequenced in the flow cell. Because SARS-CoV-2 genomes are similar, the assembly is guided by mapping all the sequenced reads to a reference sequence. A consensus of these references is then obtained as one linear sequence from a patient’s sample.

Next generation sequencing (NGS) platforms permit high-depth sequencing of nucleic acid and short-read sequencing technologies have high throughput and high-cost efficiency. Illumina sequencing platforms rely on massive parallel sequencing of short DNA strands on a flow cell surface, where they are first amplified in clusters. The cluster is sequenced using reversible fluorescent dye terminators, where the fluorescent signal is emitted at each cycle – determining the nucleotide base (or ‘sequencing by synthesis’). At the beginning of an epidemic, “shot-gun metagenomics” can be used to identify the presence of an unexpected or novel microorganism by sequencing its genetic material; i.e. using samples from patient(s) with a characteristic disease of unknown cause (Fig 2A). Hence, fragmenting the DNA (or RNA) present in a sample and sequencing it reveals new, previously unknown, nucleic acid sequence. This approach was used to determine the SARS-CoV-2 RNA virus genome sequence in samples taken from patients with pneumonia in Wuhan, China, in December 2019 [45], [46]. The same approach has been used internationally to sequence viruses from bats and diverse wildlife species, in an attempt to understand the potential origins of SARS-CoV-1 and SAR-CoV-2 pandemics [47]–[49]. Although the output from NGS sequencing are short sequence data “reads”, these are easily “assembled” by virtue of their overlapping sequences elements, meaning the data can be used to generate a contiguous (“contigs”) viral genome sequence. When new viruses emerge, the taxonomic classification and viral identification is based on this nucleic acid sequence information (Fig 2A). A known “reference” sequence is quickly established, that can be directly compared to the genome sequences of viruses detected in patients in any country (Fig 2B).

Directed, or targeted, genome sequencing approaches are more frequently used in the settings of national or global pandemics because they are considerably more efficient. In fact, in shot-gun metagenomics only a very small fraction of the sequenced nucleic acid in a sample belong to viral genomes. Most is the host cell nucleic acid and non-viral in origin. Therefore, once the viral sequence is known, targeted sequencing is used because only the viral nucleic acid is sequenced, permitting higher throughput.

**FIGURE 3.**
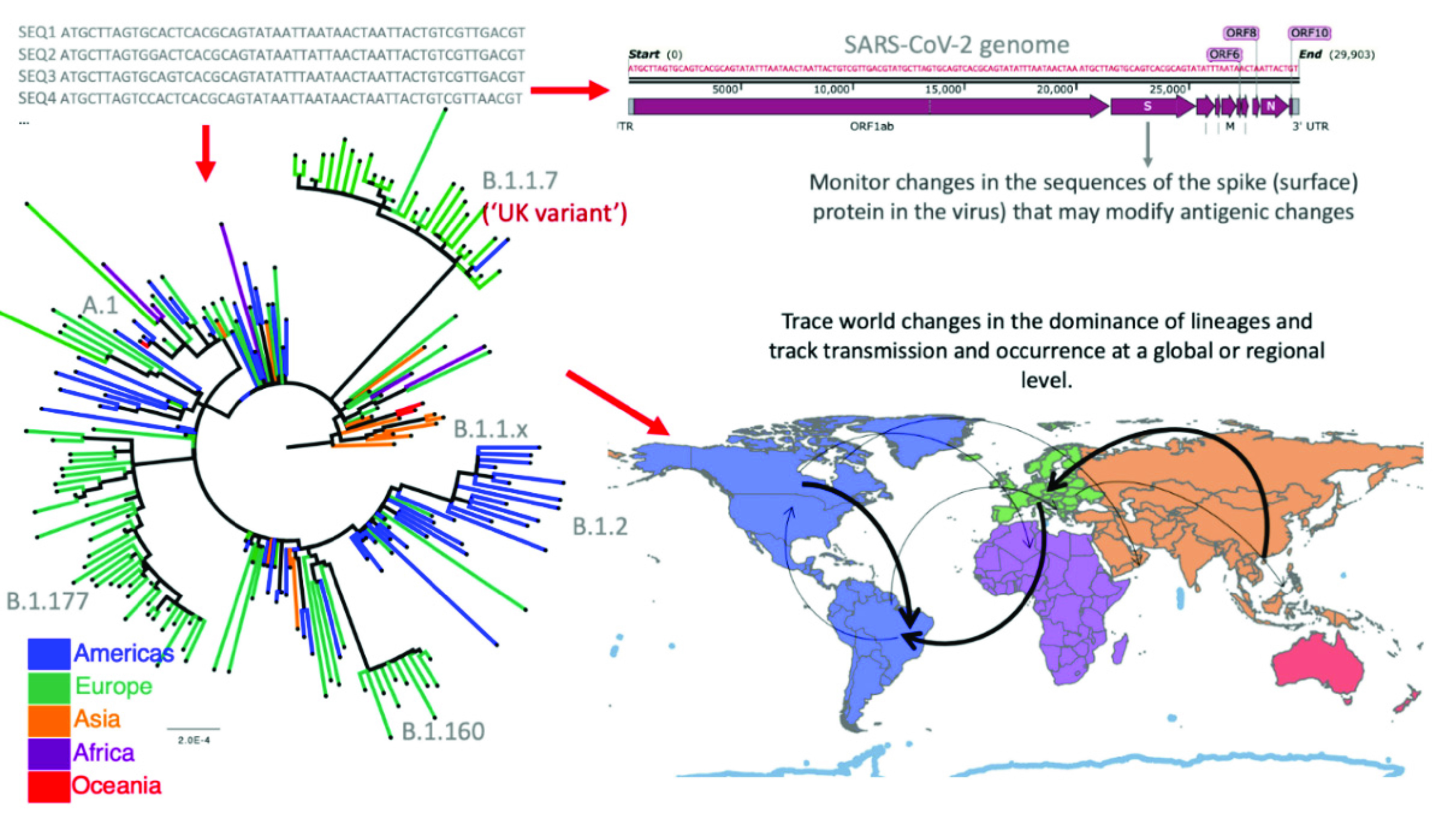
Uses of viral genetic sequence data. With the thousands of sequences obtained globally, phylogenetic trees are constructed to understand the evolutionary relationship of the viral genomes. When integrating this information with the epidemiological data (for example, the sample’s location of origin and day in which the sample is collected) maps that illustrate the virus spread and evolution can be generated. Tracking new lineages or sequence variants (viruses that are genetically related and that group in a branch of the phylogenetic tree) can help the early detection of a potentially highly transmissible or more virulent form of the virus. For example, the specific changes in a viral spike protein sequence can be monitored - the spike protein is the viral surface protein engages with a host cell receptor molecule. The viral spike is recognized by the host immune system antibodies that confer immunity. Therefore, tracking changes in the spike protein sequence identifies the evolution of the virus with respect to its antigenic properties and even the capacity of vaccines to confer immunity – protection capability. The phylogenetic tree in the image was constructed using 200 random samples from GISAID database, IQ-TREE [54] with the GTR + F + R2 substitution model. The map was created in QGIS [55] and the genome map was created using SnapGene [56].

NGS sequencing provides information about the evolution of the virus in SARS-CoV-2 coronavirus-naïve human populations. Sequence information is shared through centralized repositories such as NCBI GenBank or GISAID [50], [51]. (GISAID currently has >4.5M SARS-CoV-2 sequences as at October 24, 2021) (Fig 3A). The results then help Public Health decision-making that are implemented a health control measures. Thus, this type of sequence information can determine the index cases during specific local outbreaks, and the epidemiological spread of a specific stain of the virus. In Australia, for example, SARS-CoV-2 sequence data from local community transmission was linked to imported cases in quarantine hotels which directly led to a review of the containment protocols to better prevent community spread of incoming international strains.

The main value of genomic analyses of viruses is that it can reveal the molecular basis of emergence (and dominance) of virus variants [52]. Whilst the factors driving variant emergence remain a separate virological issue, the impact of variants ranges from potential immune escape (including a vaccine escape mutation), enhanced virulence, increased capacity for infectious spread or the development of resistance to an anti-viral drug. Viral variants emerge because viruses are inherently highly replicating organisms and replication of the genome is sometimes imprecise. Thus, genetic evolution of viruses occurs quickly, and the extend of evolution and spread is shaped by multiple host and environmental factors such as immune response competency and vaccination status. A UK viral variant, initially known as B.1.1.7 but now referred to as the ‘alpha’ variant, was first flagged in December of 2020 as a being “more fit” virus after observing that its frequency was rising much faster than other viral lineages [53]. Similarly, the delta and omicron variants are more transmissible (infectious), whereby only fleeting contact is required for human-human transmission. As a consequence, many countries are tightening public health measures based on detecting this variant within a community outbreak [7] and global access to this information can direct the option to change vaccine virus strain formulation, if/when required.

### C. Antibody Tests

“Antibody tests” provide evidence of current versus prior infection by detecting the presence of antibody molecules in the blood. An antibody, also technically known as an immunoglobulin (Ig) molecule, is a multi-chain protein comprised of two heavy chains and two smaller or light chains. The combined heavy and light chain region binds to an “antigens” with high specificity and high affinity. Other regions with the antibody molecule mediate several effector functions such as binding to Fc-receptors highly expressed by phagocytic macrophages and natural killer cells that facilitate removal or killing of virus infected cells. When a B lymphocyte produces an antibody, it first produces an IgM- antibody. It then changes its heavy chain to create another antibody molecule, an IgG, with the same specificity to the viral antigen – a process known as “isotype switching”. Hence, the antibody affinity is increased, and new Fc-dependent effector functions are gained whilst retaining specificity. Detection of only IgM tends to indicate a current infection, whereas the detection of IgG only (and low or no IgM) indicates a more distant or past infection.

Antibody molecules are soluble molecules present in the plasma component of blood and are readily detected by an enzyme-linked immunosorbent assay (ELISA). In this assay a purified protein is bound to the wells of a 96-well flat bottom plastic dish. A “blocking” step using fat milk or bovine serum albumin is employed to bind to any residual plastic surfaces not already covered by the antigen. If any virus-specific antibodies are present in-patient plasma they will bind to the antigen (present on the wells of the dish). Bound plasma antibodies are detected via the use of another or “second” antibody with specificity to the type of human Ig; i.e. IgM or IgG. The detection antibody is chemically conjugated to an enzyme, whereby provision of the enzyme substrate permits a color reaction. Results are reported as positive (color) and negative (no color), but if serum dilutions are assayed, then the amount of antibody titer yielding a positive result is quantitated by reading color intensity spectrophotometrically. Carefully performed, ELISA assays can demonstrate an increase or decrease in the amount of a pathogen-specific IgM and IgG thereby providing a temporal indictor or “infection clock”. Antibody testing therefore identifies people who have been infected but already recovered from COVID-19, by detecting the presence of virus-specific antibodies. These individuals may be eligible to donate their blood for clinical use as convalescent plasma to treat others with severe life-threatening disease [57], [58]. If required, ELISAs can be used to evaluate vaccine effectiveness by detecting the vaccine induced virus-specific antibody. Importantly, an antibody test determines the true number of cases and extent of a pandemic spread because it also identifies asymptotic cases [59]. Antibody-based tests have been developed as point-of care (POC) items for consumer use and are already available for the diagnosis of pandemic or seasonal viruses such as influenza and SARS-CoV-2 [60], [61]. Below, the current range of antibody-based POC devices and their utility with smartphone-connectivity are summarized.

#### 1) Lateral Flow Test Devices

**FIGURE 4.**
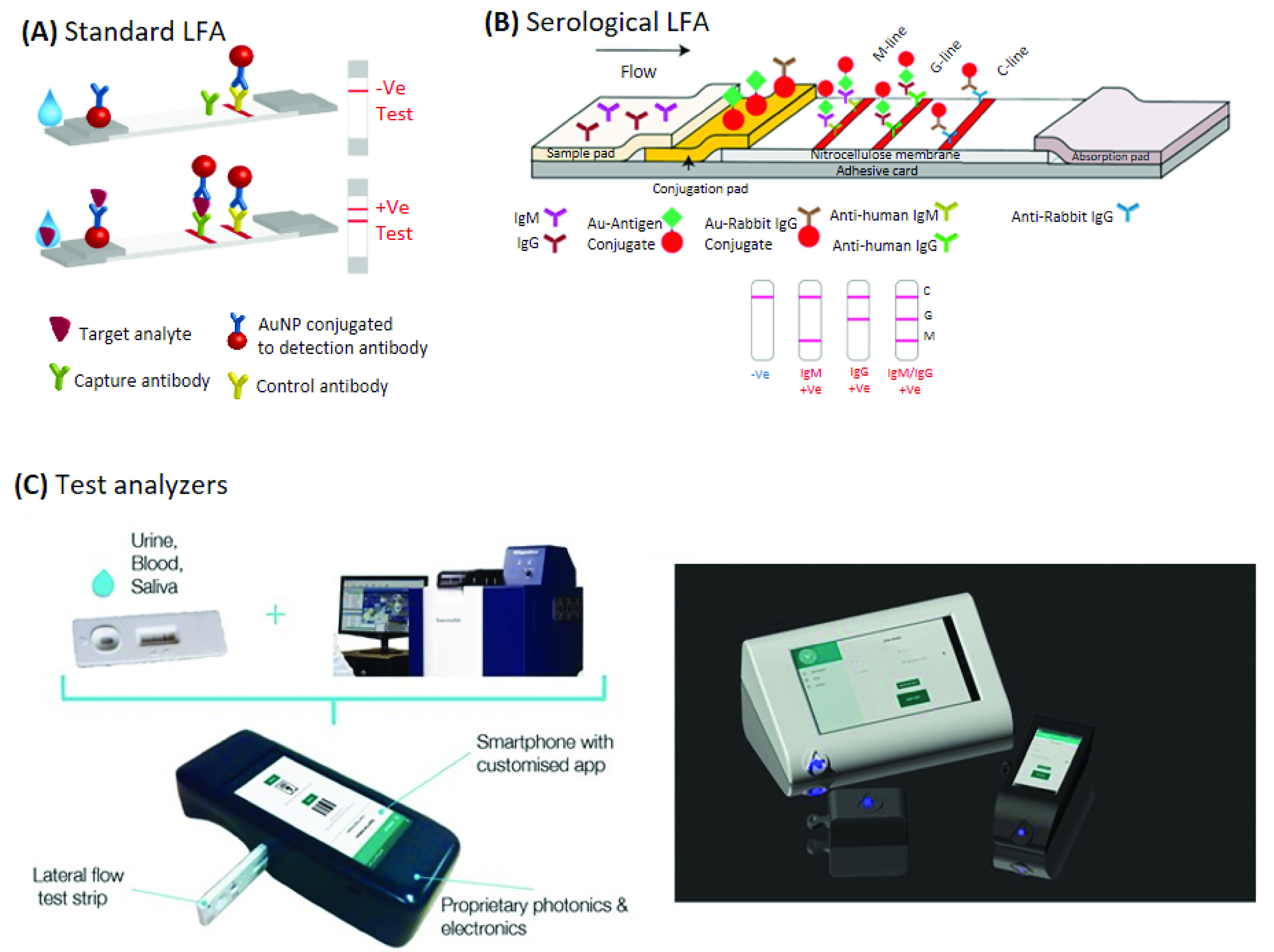
Lateral flow-based detection devices. (A), (B) General immunoassay techniques using rapid diagnostic lateral flow assay (Image reproduced from [96] permission is not required). (C) A smartphone analyzer for lateral flow tests reduces a large benchtop instrument into a portable hand carried device that automatically collects data and sends into the cloud. (Images C courtesy of AusSI Systems).

Like an ELISA, a lateral flow test device detects the presence of antibodies in plasma. It uses a paper-like membrane strip usually coated with two-lines. For example, gold nanoparticle-conjugates are present in one line and capture antibodies in the other line [62]. The patient’s plasma is drawn with capillary action across the strip (Fig 4A) and as the sample diffuses past the first line, the antibodies bind to the gold nanoparticles and the conjugate flows through the membrane. As it reaches the second line, the conjugate is immobilized by the enzyme-conjugated capture antibodies, and the line becomes visible after substrate is produced. Individual gold nanoparticles are red, but a solution containing clustered gold nanoparticles is blue (due to coupling of the plasmon bands). Appearance of the color indicates a positive test, while an additional color line (control line) confirms the validity of the test (Fig 4A & B). These assays are only semi-quantitative as determined by the color intensity. The main disadvantage is operator variability: a color-limited vision person could find distinguishing the color difficult and the test is unusable by a blind person. However, the test is ideally amenable to smartphone analysis (removing this operator variability). Lateral flow “rapid diagnostic tests” (RDT) can be performed under 30 minutes. Nearly 40% of all the FDA-approved antibody tests developed within the first six months of the SARS-CoV-2 pandemic are rapid antibody tests [63]. More importantly, these type of test results are highly concordant with laboratory ELISA assays [64], [65].

Smartphone-based lateral flow device detection methods already exist (Fig 4C) that enable instant communication of test results to Public Health officials and rapid global data collation via cloud-based systems [66]–[68] (see Section V). This permits rapid deployment of health measures to targeted areas. The value of these technological capabilities are immense in rural areas were scientific laboratories may be absent or extremely distant from the source of the outbreak.

#### 2) Microfluidics Assays

In a microfluidic device, the color is produced in a reflecting path on a transparent substrate to improve signal-to-noise levels. Some complex microfluidic structures make use of 3D-printable transparent polymers such as polymethyl methacrylate (PMMA) [69]. The digital assay applies an electromagnetic field to manipulate and mix individual droplets on a superhydrophobic plate (reducing the risk of physical contamination). For low-cost and flexible operation outside of the laboratory, paper-based analytical devices can be more suitable than polymer-based microfluidic chips [70]. Multiplexed detection of antibody response from different antigens of SARS-CoV-2 has been achieved with microfluidic POC tests [71].

Issues of false-positive or false-negative test rates remain, with the primary disadvantage being related to sensitivity of the small sample quantity being tested. This small volume confers several limitations. For example, when a low virus (antigen) sample is loaded, the antibody detection molecule may never interact with it. Also, if allowed to “float” within the sample chamber, there is a statistical hit and miss ratio that worsens with decreasing concentrations or with increased volumes. Issues of sensitivity are addressed by amplifying the signal using fluorescence and chemiluminescence [75], [76], but these require excitation from laser or broad-spectrum light. Although detection requires an excitation light source, an emission filter, and photodiode or CMOS chip, highly-sensitive tests have been developed for the fluorogenic detection of antibodies specific to zika virus (ZIKV), chikungunya virus, and dengue virus (DENV) using a two-stage LAMP-based microfluidic nucleic acid detection [72]. The limit of detection is currently at the level of single copy of nucleic acid in PBS and 10 copies of viral genetic material in human serum and the sensitivity can be increased by using 3D membrane filters capable of rapid and multi-fold analyte capture [72], or simply delivering more capture antibody. Electrochemical biosensors are also being developed that produce an electrical signal or an optical emission (electroluminescence). The electrical signal is directly plugged into a portable display device, or a camera can be used to detect the luminescent output, and data is digitized. Gold electrodes functionalized with self-assembled thiol chemical groups have been used to detect ZIKV DNA in a device that measures impedance changes [73]. Electrode capacitance has also been used to detect DENV antigen-antibody complexes [74].

## IV. Smartphone-Based Rapid Diagnostics and Other Emerging Technologies

**FIGURE 5.**
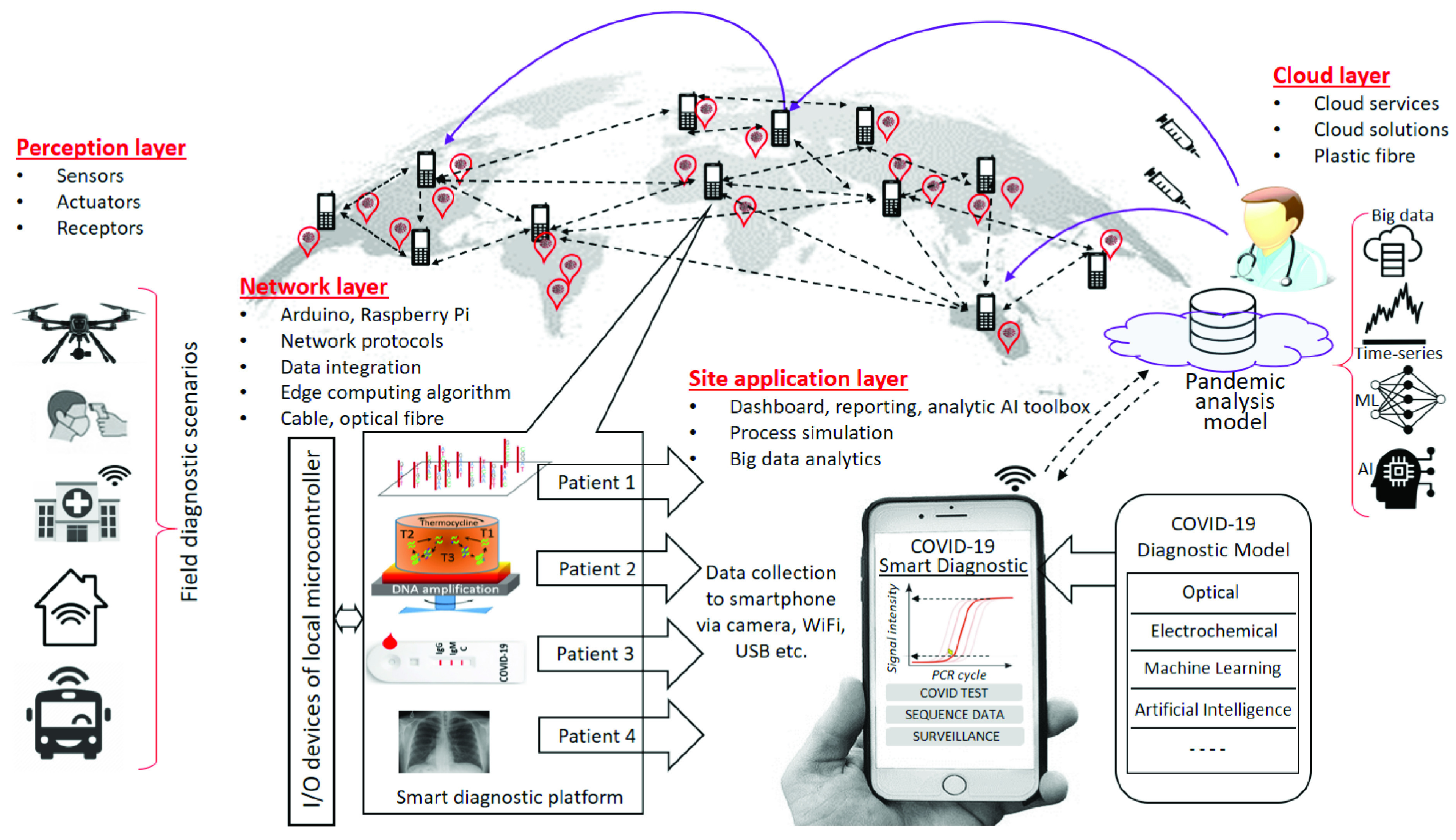
Transmission networks underpin the internet and cloud-based diagnostics and informatics capabilities. Diagnostics at the perception layer feeds into the cloud via the edge using various transmission capabilities such as wireless and optical fibers. Alternatives exist for how data can be transferred. The results are collected at a central server from detection sites by the sensor devices which are used to analyze sequence, predict virus types using AI and develop a suitable vaccine which can be prescribed to the affected sites.

During infectious disease pandemics, rapid testing delivers the means by which infected people are identified without overwhelming a clinical laboratory’s capacity for traditional benchtop testing. A key limitation with any rapid testing is interpretation of the result and its associated errors. There is therefore a need for real-time testing and in-field tracking that links cloud-based analysis and diagnosis messaging. Given the widespread global prevalence of smartphones, this would be considered as the current technology platform of choice. The smartphone itself acts as the diagnostic instrument whilst simultaneously transmitting and receiving data instantly to cloud-based databases and software [75]. This approach allows instant disease identification and notification locally, nationally or globally via mobile networks (Fig 5).

### A. Smartphone Based Nucleic Acid Tests

Components for a fully integrated field device already exist for sample collection, nucleic acid extraction, amplification and signal processing. For example, extraction of nucleic acid (from respiratory samples) can be driven by low-power fans mounted onto the smartphone, providing centrifugal force-based extraction [76]. They can also be used in an in-flight drone centrifuge and convective PCR run from a smartphone [77]. The nucleic acid test can be performed based on Rayleigh–Bernard natural convection for buoyancy-driven thermal gradients of liquid heating [78]–[80]. Heating occurs from the bottom of the reaction, meaning a spatial temperature gradient established between the top (cool) and the bottom (hot) surfaces of the reactor, simulating a thermocycler. In other words, the movement of solutions by convection physically transports reagents between reaction zones. Renewable energy sources such as solar and body generated thermal heating can also be used [81], [82]. Self-contained platforms have been reported for smartphone devices that perform both temperature tuneable and time-resolved fluorescence measurements with continuous photographic and self-controlled timing of the detector [83], [84]. There are also reports of rapid isothermal amplification for COVID-19 [85], [86]. In another example, a hot coffee mug is used to heat the RT-LAMP reaction for colorimetric detection of ZIKV RNA [87]. The device uses an innovative ball-based valve to enable the storage and delivery of reagents in a paper-based system within the mug for RNA enrichment and purification. In another design, water-triggered exothermic materials offer a low-cost SmartCup energy efficient reaction [88]. Additionally, LAMP reactions are possible on microfluidic paper integrated with a smartphone fluorescent reader [89]–[92]. Moreover, multiplexing can achieve simultaneous reactions of different viruses [92]–[94], and indeed, a silicon microfluidic chip containing 10 parallel flow channels has been developed for multiplexed LAMP detection of bacterial and viral pathogens where a smartphone camera is used to capture the fluorescence readouts [92]. Finally, although analysis of the NGS reads is generally performed using high-end server computers or cloud services requiring massive machine to machine data transfer through a broadband network, MinION is a compact portable devices that use similar technology allowing sequencing in the field [95]. Edge analysis greatly reduces bandwidth within an IoT network. Alternatively, bioinformatic analyses have been directly undertaken on the smartphone. This includes sequence alignments to detection of variants [97]. Near-real time sequence data analyses greatly enhance clinician and epidemiological data tracking [98] for deployment of optimal treatments.

### B. Smartphone-Based Antibody Tests

The digitization of the antibody colorimetry, albeit on a test strip or an ELISA plate, has long been part of measured outputs [99]–[102]. Several companies are aiming to develop single-use, disposable serological testing for SARS-CoV-2 antibodies using smartphone devices [103]. Typically, the assay integrates smartphone camera imaging for rapid digital quantification of dot-based ELISA tests [104]. Early assessments suggest these rapid assays can match laboratory grade accuracy. Further, 3D-printing technology has been used to fabricate packaging and customized optics for multiplexed ELISA detection using smartphone cameras [105]–[107]. One drawback of smartphones as a diagnostic is that they do not have a consistent color correction, particularly for multiple color parameters, and color detection varies across models [108]. Nonetheless, individual smartphones can be calibrated and performances comparable with benchtop equipment. There are ample examples available with smartphone-based detection. Smartphone detection for avian influenza specific human serum antibodies has been achieved with a sensitivity of 96.5% and a specificity of 98.5% [109]. In another example, a smartphone camera images fluorescent labelled secondary antibodies in barcode-like patterns upon the recognition of the virus [93]. Here, the optics for excitation requires an additional external power source (typically, a monochromatic light source requiring a heavy power supply or rechargeable battery). This can limit POC portability. For some cases, however, it is possible that an excitation source can be powered by the smartphone in-built rechargeable battery or a USB connection to an external hard-wired power supply [110], [111]. Furthermore, electrochemical biosensors generate an electrical signal output or a luminescent emission [112]–[116]. The output electrical signal can be directly plugged into the smartphone via its micro-USB port and a luminescent output can be detected via the camera with subsequent digitizing of the output information. Several microcontroller platforms have delivered affordable electrochemical biosensors this way [112]. A geno-sensor device has been designed for the differential detection of ZIKV infection and its discrimination from other human flavivirus infections such as dengue virus [113]. In this example, the sensor captures signal probes and the PCR amplifies the target genetic material. Similarly, an electrochemical impedance sensor imprinted onto a traditional lateral flow strip has been reported for portable quantitative detection of ZIKV [114]. The impedimetric sensor uses a gold micro-electrode antibody-sensing chip – prepared via immobilization of ZIKV specific envelope protein antibody [114]. A portable platform has been developed to perform some of the more common electroanalytical techniques of chronoamperometry, differential pulse voltammetry and square wave voltammetry [115]. There are in fact a broad range of exciting new technologies being developed, in addition to smartphones, for rapid testing applications including SARS-COV-2 diagnosis. These are summarised below and in Table 1.

**TABLE 1:** Innovative Technologies for Detection & Analysis of Infectious Diseases

Method	Target	Prospects	Constraints
Section A: Nucleic acid testing			
Smartphone RT-qPCR	DENV DNA [132] Avian influ. DNA [79] Salmonella DNA [133] Human 18S ribosomal RNA [134]	• Digital quantification makes multiplexed detection and identify co-infections • 3D-printable, auto sample injection and control • Smartphone processing and reporting, faster than clinical diagnosis (< 1 hr)	• Thermal instability, power requirement and complex • Need interfaces of genome collection and extraction • Dependence of viral load
Smartphone convective PCR	H1N1 influenza-A RNA [79] S. aureus DNA [77], [80] E. coli O157:H7 DNA [76]	• Buoyancy driven natural convection • Less power and electronic control • On-board genome extraction using miniature fan/drone-based centrifuge	• Open-loop temperature control • Lack of integrated sample preparation capability
Solar thermal PCR	Kaposi’s Sarcoma herpesvirus DNA [81]	• Solar heating for PCR thermocycling • Low power (80 mW), fast response (<30 min) • Smartphone based fluorescence detection	• Limited lifetime (phone battery) • Temperature adjustment needed for weather variation
Smartphone LAMP	ZIKV RNA [87] Herpes simplex virus DNA [88] SARS-CoV-2 RNA [86] Influenza-A H1N1, S. aureu [85] P. falciparum DNA [89] HIV RNA; EHV1, EHV4 and EIV DNA [92]; ZIKV, ENV, CHIV RNA [90]	• Fixed and relatively lower T requirement • Multiple primers increase specificity • Innovative designs of reaction chamber (e. g. SMART cup/mug) allow storage and sequential delivery of reagents, RNA enrichment and purification, exothermic chemical reaction • Fast POC test such as with smartphone set up (<40 mins)	• Need an RNA extraction kit • Multiple primers and regent costs
CRISPR	crRNA of SARS-CoV-2 [41]	• Ultra-sensitivity, specificity and accuracy • Colorimetric and fluorescence readouts • Capable of handling many samples at a time • No need of RNA separation and amplification	• Limits uses at the patient’s end as it needs skilled operators
Section B: Antigen or Antibody Testing			
Smartphone immunoassay tests (LFA, ELISA, ECL)	HIV/syphilis IgM /or IgG [135] Influenza-A [104] Avian influenza H5N3, H7N1 & H9N2 [109] CD4 test of HIV [136] Type-I pyrethroids in pesticides [137]	• Low-power, ultra-portable with low-cost PAD • On-chip sample collection and purification with less hardware, user intervention and regent cost • Space and wavelength division multiplexed detections using smartphone optical readout • Rapid detection (10–30 mins) including asymptomatic carriers, site identification, geographical transmission and past infection • Comparable to clinical ELISA: sensitivity >90%) specificity >80%; accuracy >95%	• Does not always show active infection • Variation of antibody levels: suffers sensitivity issues at early during an infection • Misleading results when cross-reactivity of antibodies specificity is evident • Many are yet to show effectiveness in COVID-19
Section C: Other emerging diagnostic technologies			
Plasmonic photothermal biosensing	SARS-CoV-2 RNA [119]	• Ultra-fast optical detection (6–10 minutes of viral RNA hybridization) • Specific and sensitive detection reduced false negative results	• High precision optics alignment • Statistical hit-and-miss as interaction area are small which can reduce sensitivity
FET-based nano-biosensing	SARS-CoV-1 RNA [138]–[140] Ebola VP40 matrix protein [141]	• Low-cost, label-free detection • On-chip fabrication, wearable device • Novel functional material (graphene, silicene etc.)	• Packaging and stability are challenging issues • Need electrical isolation
Optical interferometry	SARS-CoV-2 RNA/Antigen [142]	• Ultra-fast optical detection (6–10 minutes of viral RNA hybridization) • Reduced false negative results • Demonstrated for SARS-CoV-2 detection	• Packaging and stability are difficult
AI-based imaging	Patient lung image [128], [143]	• Portable -ray, CT, digital microscopy tools • Advanced computer vision, machine learning algorithms improves detection accuracy • Smart health system management and control	• Insufficient data to train algorithm • Security, privacy issues • Low specificity of imaging tests.

Most recently, an exciting new avenue in this space has been demonstrated. The detection of three chemical samples reference against a reference sample within a novel, rugged smartphone spectrometer is further evidence of what technology can achieve in simultaneous near real time measurements into the network [117], [118]. The potential to analyze multiple parameters and samples simultaneously in the field can only support and enhance the capability of a connected super diagnostic network. It illustrates the power and potential packed into the everyday consumer smartphone, a technology that is also expanding in other consumer products that will also help change the landscape. What also arises form this is the importance of pre-processing edge computing within the smartphone to help reduce the bandwidth demanded by the network.

### C. Optical and Quantum Technologies

A number of photonic based methods are being applied to sensing and diagnostics. For example, a localized surface plasmon resonance (SPR) biosensor was reported using 2D gold nano-islands making up the plasmonic material and functionalized with cDNA probes as a sensor for SARS-CoV-2 [119]. Nanoparticles within the surface plasmon excitation region have broad optical cross-sections coupled to non-radiative decay that produce highly localized heat upon optical absorption. This heat is adjusted to make it more difficult for imperfectly matched sequences, compared to the desired target, thereby reducing false-positive readings. In another SPR example, a metal-coated optical fiber generates a propagating plasmonic surface wave at the interface to measure changes in the refractive index of the environment being analyzed [120], [121]. Here, the refractive index changes with a virus antigen and antibody interaction. The level of sensitivity of the detection can be improved by using 3D-printed novel fibers that increase surface interactions with the analytes [122], [123] as well as by using other fiber optic resonant components such as Bragg gratings [124]. Highly sensitive detection can also be achieved using biomedical waveguide interference between two optical modes [125]. The visible light interacts with analytes during propagation and the output interference is recorded using a photodetector array. The optical detection of SARS-CoV-2 coronavirus can be accelerated by combining the magnetic affinity of nanoparticles into the detection system [126]. This technique uses the virus RNA specific fluorescent probe that attaches to the viral RNA and emits light when excited by a laser at an appropriate wavelength. With the use of magnetic particles that adhere to the fluorescent molecules, a magnetic field can drive their movement through the solution leading to aggregation in the illumination area of the laser beam, helping to amplify measured signal strength. This technique can potentially detect ultra-low viral RNA concentrations [127].

Optical imaging modalities (chest X-ray and CT scans) were initially used to diagnose the COVID-19 infections in patients in Wuhan hospitals [128]. The obvious issue here is that CT scans are nonspecific with respect to an infectious agent, although they can illustrate a certain pathophysiology and identify their anatomic location. In situations where clinicians are unavailable, such as in rural locations, or even in overly busy central hospitals, machine learning possibly with AI, might triage the incoming data to categorize patients relative to the limited available resources in pandemic settings [129]. The initial challenge is access sufficient realistic clinical data for algorithm training and to test and verify high accuracy.

A significant limit to all testing is noise and, more specifically, shot noise limits that arise from the quantized particle nature of electronics and light in sources and detectors. When considering photon emissions from a source, the electronic transition is random in time, leading to fluctuations. In the classical domain, where many photons produced as an average, this is not detected except when the photon number becomes small. However, in the case of a diagnostic that needs to detect only a few photons (i.e. for single particle detection) then noise can limit detection reliability. Consequently, to get below the shot noise, control of emission of the photons is required and typically achieved using single photon emitters. Currently nitrogen vacancy centres in diamond are being used [130] and these have been applied to the detection of label-free DNA in microfluidic devices [65]. Small photodetectors optimized to reduce the shot noise can be designed. However, the detection of single photon emission itself requires correlation with a second single photon source [131]. The centres that make good single photon sources tend to have high quantum efficiencies which leads to higher intensity emission.

### D. Nanomaterials and Nanotechnologies

Nanomaterials, such as graphene or silicene, within nanodevices such as field-effect transistor-based transducers, can be used as bioreceptors for antigen or antibody, delivering high sensitivity [140]. These materials can act as the channel within a fluid environment and also as a liquid gate - where the channel material surface is attached with a sample under test [138], [139], [141]. Both a SARS-CoV spike glycoprotein-specific antibody, and the ACE2 receptor protein have been crosslinked to graphene [138], [139], [141]. When integrated as channels within isoporous membranes this approach achieves high affinity detection [144].

Nanomaterial porosity offers a filtration function, particularly when integrated in novel formats, and self-assembly fabrication reduces cost, producing bulk amounts of high uniformity [130], [145]. Such structures have been identified as suitable in optimizing hydrogen generation [146]. These nanomaterial wires promise a new generation of diffusive strips that could outperform current paper-based approaches that rely on randomized pore sizes that limit the resolution of lateral flow tests. This is a rapidly developing area and future innovations are certain to significantly disrupt POC diagnostic test capabilities.

### E. Wearable Sensors and Smart Fabrics

Current technologies for wearable sensors integrate nanomaterials for a variety of functional readouts with applications ranging from sports and endurance monitoring to health readouts. For example, thermal sensing fabrics can be produced by incorporating conductive polymers, graphene, nickel, silver and copper metal nanoparticles and nanowires (for recent reviews see [147] and [148]). Currently, multi-modal biosensors are expected, and they typically include temperature, pressure, and biomaterials for detection and monitoring excretions and metabolites. These wearable sensors can be embedded onto human skin or prepared within worn human devices, even fabrics or items such as mouth guards. They typically monitor heart rate, blood pressure and/or metabolites. Wearable devises monitoring body temperature (symptoms of fever) [149] and blood oxygen levels are particularly relevant to COVID-19 where hypoxia is a typical presentation of SARS-CoV-2 infected symptomatic patients with pneumonia. Additionally, automatic alarms can be built-in. These can, for example, be used to alert when temperature thresholds are reached.

Smart textiles design and treatments with anti-viral agents can create an invisible coating that inhibits viral sustainability. For example, it was mentioned earlier that known anti-virals can be inserted into face masks. Coatings that can repel liquids and potentially prevent viruses from adhering to the surface have also been demonstrated [150]. It has long been known that silver-based technologies can destroy enveloped viruses including coronaviruses by 99.99% [151] and, using this, a “disinfectant velvet” has been developed that is capable of photocatalysis when exposed to UV light [14]. Silver ions in various formats are impregnated into material for face masks for their anti-viral properties. These technologies are rapidly expanding, and although clearly exciting, a degree of caution is required because the degree of accuracy of these detection fabrics, and the limits of sensitivity are yet to be properly evaluated. Yet a highly infectious person might one day be both contained, detected and identified using masks comprised of smart fabrics.

## V. Network Diagnostics of Diseases and Real-Time Bioinformatics Capabilities

Smart diagnostic tools include smartphones, smart-watches, smart cloths, wearable devices and smart body implants with bio-receptor elements and smart functional materials. These tools can allow large-scale collection of patients’ information in real-time. This allows aggregation of “big data” compiled and processed in real time via global IoT networks. Such an advance will mark a new generation of disease “network diagnostics” where monitoring is powered by “swarm” artificial intelligence (AI) arising from the use of millions of field devices taking advantage of federated learning where an algorithm is trained across multiple decentralized smartphones or other devices operating at the network edge and servers where sample data is stored. In effect, the diagnosis happens at the patients’ ends. The information is coordinated and distributed for use directly, nationally or even internationally. Communication can be two-way with interconnected sensors being capable of communicating to each other, as well as centrally, using multi-layered architecture communication networks for both transmission and reception.

*The perception layer* is the physical layer, where sensors collects information at the point-of-interest (i.e. the patient at their location) and processes the information. Smartphone camera sensors are customarily used as colorimetric and fluorescence detection in many PCR, LAMP and various rapid tests devices for virus nucleic acid or antibody/antigen detection (discussed earlier summarized in Table 1). Multiple diagnostics can be undertaken at patients’ sites where the location information for mapping and tracking can be collected through GPS and other positioning sensing approaches. For example, heat detection of people with fever, individually or within small or large groups can be mapped. It can be individual-based or crowd-based sensing connected made possible via a dedicated sensor network. Distributed sensors can be positioned to monitor within surrounding infrastructure.

*The network layer* involves automated data collection. Conventionally, this is achieved through edge sensors that transmit data to wireless base stations and from there via optical fiber into the cloud where the applications layer resides. “Smart” platforms largely deployed in the perception layer can use a wide range of communications such as Bluetooth, LoRaWAN, Li-Fi, local 5G, Wi-Fi, GSM and satellite including localization (GPS). Whilst the smartphone is typically transmitting in RF or mm domain (from 3G to 5G, and by ~2030 6G, depending on location), the sensor itself may be decoupled and transmit data to the smartphone and other mobile base units (MBU) via various protocols. Examples currently used in the medical space include acoustic and Bluetooth supported by Arduino, Raspberry Pi, Beaglebone Black and others. All these methods used in any arbitrary number of devices can be integrated together via the IoT, and when coupled with federated learning, be integrated directly together for simultaneous global mapping and more.

*The application layer* includes application specific software and database analysis. Data from the previous levels are stored, aggregated, filtered, processed and analyzed. Once integrated, all the tools of analysis, from data collection to machine learning (ML) and AI derived decisions that expedite turnarounds, can be brought to bear both at the edge and across the cloud that describe much of the IoT infrastructure. For example, simply linking automation and rapid diagnostics permits comparisons to pre-existing reference data including virus nucleic acid genome sequences, instantaneously via cloud connectivity. This increases the value of the data by reducing translation times and reducing the potential for data errors. These communication networks allow for direct linkages to be made in all directions, simultaneously. They offer a new paradigm for immediate disease tracking and control. Global ubiquity can theoretically provide equal global accessibility, an important social aspect that may prove more challenging to resolve than the technology.

Real-time communication permits quick translation of information that can deliver optimal treatment – basically, real time tracking compared to that currently used for COVID-19. For example, specific diagnosis of a distinct variant irrespective of the location of any infected patient is feasible as illustrated in Fig 5. The general concept has been virtually tested in a simulated respiratory virus epidemic already with the British Broadcasting Corporation documentary *“Pandemic*” that describes a situation where a smartphone application was employed in a hypothetical 2018 new influenza epidemic as part of a “citizen science” experiment. 28,965 people in the UK voluntarily provided their contact and movement data from their smartphone (collected by GPS). This was transmitted to a central server and applied in a time-series analysis to determine the incidence cases and the origins of the infection [98]. Two different scenarios were studied: (1) the start of the outbreak within a local town (Haslemere in Surrey; within-patch), and (2) the spread of the pandemic through the UK (between-patches). The analysis used a discrete-time SIR (susceptible people – infectious – recovered) model to determine the output parameters. It considered the user profile, their movement, encounter times, relative transmission co-efficient and reproduction ratio [152]. Although this analysis did not explicitly include the mortality rate, it did successfully demonstrate how the extra control measures can considerably slow down an infectious outbreak to reduce its impact [152]. Improving on this, individual sensors can be embedded within this network, providing Health Officials with real-time diagnostic tracking information and location mapping. That would enable near-instant targeted response containment, control and treatment measures.

As with all informatics the utility of the data is highly dependent on the quality of the direct measurement. Machine learning or AI can be an invaluable tool for accelerating and automating analysis with increasing accuracy – whether it be to filter out the negatives or to positively identify different type or stages of infection cases [129], [153]. Quickly screening and removing negative test results builds resilience to testing errors. An intelligent global network can facilitate the reduction of individual testing, otherwise needed, by utilizing full statistical analysis to identify and isolate significant errors across the measured ecosystem as a whole. Already, a neural network framework through deep learning has shown a COVID-19 prediction accuracy of 82% using smartphone sensor measurements [154]. This will likely increase once these approaches are trialled more broadly and frequently. Whilst bioinformatics is still in its infancy, the spread of smartphone detection instrumentation and edge processing, coupled with swarm technologies, means implementation of mass data analytics in real-time is near and highly likely to be the employed early in future global pandemics. The quality of guaranteed data transmission, or connectivity, is another current concern since not all countries have extensive or modern network infrastructure. This problem is familiar to those in IoT where regional application of technology in rural settings even within developed countries have unreliable connectivity. Nonetheless, demonstrations of current capabilities exist: US FDA approved heart ECG monitors that function well, including across ocean distances over time, are commercially available [155].

At present, however, the need to include secure and equitable data transmission and an analysis that is accountable to national and globally agreed standards cognisant of accepted ethical standards are political and social challenges that need addressing. Exactly “what” and “how” data is to be shared - across state or country borders – and the issue of ownership of the electronic data, must be addressed imminently. Indeed the WHO has recommended that a global diagnostics internet be centralized and administered by a universally accepted authority [156]. Thus, the global COVID-19 pandemic has already begun to accelerate these types of technology uses – whether the political and social will is there to connect the world fairly remains to be seen.

## VI. Conclusion

This paper presents a cross-disciplinary review on pandemic respiratory virus detection, particularly SARS-CoV-2. It focuses on the transmission mechanisms, detection methods, smart POC testing and connected network-based informatics. We discuss the nature of surface water, how it exists, and interacts as aerosols and droplets, in a manner that permits virus infection transmission, an issue that has largely not been considered previously but which offers practical solutions for treatment and detection. We review the laboratory and POC possibilities for virus detection. Finally, the review embraces a range of new and emerging smart diagnostics and sensor tools, powered by lab-in-a-phone type technologies, and internet connectivity, for real-time detection and monitoring with global-cloud data accumulation and utilization by Health Officials. This real time detection need not be confined to one target only, with recent advances demonstrating multiple analyte measurements performed simultaneously that can be integrated into this network. Given the potential of this technology across sectors, a challenge will eventually arise from bandwidth demands and a need to practically balance edge and cloud computing, storage and analysis to optimize connectivity differences across and within regions. These ongoing developments are unlikely to fully replace gold-standard laboratory PCR-based nucleic acid testing because it too will be integrated into the network serving as benchmark reference points for all analysis. A key advantage of these networks will be near real time diagnostics and potential resilience in mass diagnostics with lower quality assays and other tests. They will also reduce error from current, overwhelmingly manual, sample collection. Therefore, the test of choice in the future will be more about those tests that can be integrated in volume, into a hardware driven real-time informatics network diagnostic, where increasingly edge computation, as well as cloud analytics relying on collective data. As is abundantly evident with COVID-19, pandemics are global, spread through numerous vectors and environmental factors. Therefore, a global “super diagnostic” that has not yet been available, may emerge - a global network “super diagnostic” instrument capable of identifying disease and/or monitoring infected individuals, so as to enhance Public Health outcomes.

## Author Contributions Statement

Md Arafat Hossain and John Canning conceived the manuscript.

Md Arafat Hossain and John Canning performed the research.

Md Arafat Hossain, Barbara Brito-Rodriguez, Lisa M. Sedger, and John Canning co-wrote the manuscript.

Lisa M. Sedger and John Canning are equal senior authors.
